# Use of simple clinical and laboratory predictors to differentiate influenza from dengue and other febrile illnesses in the emergency room

**DOI:** 10.1186/s12879-014-0623-z

**Published:** 2014-11-25

**Authors:** Shi-Yu Huang, Ing-Kit Lee, Lin Wang, Jien-Wei Liu, Shih-Chiang Hung, Chien-Chih Chen, Tzu-Yao Chang, Wen-Chi Huang

**Affiliations:** Department of Emergency Medicine, Kaohsiung Chang Gung Memorial Hospital, Kaohsiung, 833 Taiwan; Division of Infectious Diseases, Department of Internal Medicine, Kaohsiung Chang Gung Memorial Hospital, Kaohsiung, 833 Taiwan; Department of Pediatrics, Kaohsiung Chang Gung Memorial Hospital, Kaohsiung, 833 Taiwan

**Keywords:** Influenza, Dengue, Other febrile illnesses

## Abstract

**Background:**

Clinical differentiation of influenza from dengue and other febrile illnesses (OFI) is difficult, and available rapid diagnostic tests have limited sensitivity.

**Methods:**

We conducted a retrospective study to compare clinical and laboratory findings between (i) influenza and dengue and (ii) influenza and OFI.

**Results:**

Of 849 enrolled patients, the mean time between illness onset and hospital presentation was 1.7, 3.7, and 3 days for influenza, dengue, and OFI, respectively. Among pediatric patients (≤18 years) (445 influenza, 24 dengue, and 130 OFI), we identified absence of rashes, no leukopenia, and no marked thrombocytopenia (platelet counts <100 × 10^9^ cells/L) as predictors to distinguish influenza from dengue, whereas rhinorrhea, malaise, sore throat, and mild thrombocytopenia (platelet counts 100-149 × 10^9^/L) were predictors that differentiated influenza from OFI. Among adults (>18 years) (81 influenza, 124 dengue, and 45 OFI), no leukopenia and no marked thrombocytopenia distinguished influenza from dengue, while rhinorrhea and malaise differentiated influenza from OFI. A diagnostic algorithm developed to distinguish influenza from dengue using rash, leukopenia, and marked thrombocytopenia showed >90% sensitivity to identify influenza in pediatric patients.

**Conclusions:**

This study identified simple clinical and laboratory parameters that can assist clinicians to distinguish influenza from dengue and OFI. These findings may help clinicians diagnose influenza and facilitate appropriate management of affected patients, particularly in resource-poor settings.

**Electronic supplementary material:**

The online version of this article (doi:10.1186/s12879-014-0623-z) contains supplementary material, which is available to authorized users.

## Background

Influenza is one of the most common infectious diseases worldwide [1]. The spectrum of clinical manifestations ranges from mild-form nonspecific febrile illness, such as cough, sore throat, headache, rhinorrhea, malaise, and muscle ache, to respiratory failure and death [2]-[4]. Its symptoms and signs can be similar to other viral illnesses such as dengue, making the infections difficult to distinguish. The clinical manifestations of dengue illness vary greatly, ranging from a mild, flu-like, and self-limited febrile illness to severe dengue [5],[6]. Influenza and dengue overlap geographically in tropical and subtropical regions of the world [1],[7]. Concurrent influenza and dengue outbreaks have been reported [8]-[11]. The overlapping clinical features of influenza and dengue created clinical diagnosis and management challenges during simultaneous influenza and dengue outbreaks in Puerto Rico in 1977 [11]. Dengue epidemics have occurred in Taiwan for decades [12],[13]. An outbreak of 2009 pandemic H1N1 occurred in Taiwan after the first case was identified on May 20, 2009 [14]. The overlapping symptoms and signs of influenza and dengue created a confusing clinical situation and challenges in etiology identification. Although rapid diagnostic tests can help confirm an influenza diagnosis, their sensitivity ranges from 40-70% depending on the day of illness and specimen type [15]. Definitive diagnosis of dengue is made using serology tests, but these tests are not always readily available in most clinical laboratories [7],[16]. Our study aimed to identify clinical and laboratory features that distinguish influenza from dengue and other febrile illnesses (OFI) in dengue and non-dengue endemic settings. We applied decision tree analysis to our dataset to discriminate patients with influenza from those with dengue. Our findings may be valuable for clinicians working in crowded emergency rooms (ER) in countries with limited medical resources.

## Methods

### Ethics statement

The study was approved by the Institutional Review Board of Kaohsiung Chang Gung Memorial Hospital (KSCGMH) (Document no. 102-4695B). Informed consent was not required as the data were analyzed anonymously.

### Study population, diagnosis and definition

We conducted a retrospective study of 849 febrile patients (ear temperature ≥38°C) who presented during 2008 and 2010 with possible dengue and influenza infections to the ER at KSCGMH, a 2,600-bed primary care and tertiary referral medical center in southern Taiwan. All patient medical records were reviewed, and clinical and laboratory data at the time of hospital presentation were extracted for analysis.

Patients with influenza infections were defined as those laboratory-positive for influenza with influenza-like illness. Influenza-like illness was defined according to World Health Organization (WHO) guidelines: fever, cough, or sore throat [17]. Laboratory diagnosis of influenza was made for patient's respiratory specimens (nasopharyngeal or pharyngeal swabs) positive for virus-specific ribonucleic acid by real-time reverse transcriptase-polymerase chain reaction (RT-PCR) (TAIGEN Bioscience Corporation, Taiwan) [18]. All PCRs were performed at the central testing laboratory of KSCGMH using the standard real-time RT-PCR influenza procedure described by the Centers for Disease Control and Prevention [19].

All dengue cases included in this study were confirmed by at least one of the following criteria: (i) positive dengue virus-specific real-time RT-PCR (QuantiTect SYBR Green RT-PCR kit; Qiagen, Hilden, Germany) [20], (ii) positive dengue virus-specific immunoglobulin (Ig) M antibody enzyme-linked immunosorbent assay of acute-phase serum, after excluding cross-reactions to Japanese encephalitis virus [21], (iii) a fourfold increase in dengue virus-specific IgG antibody in convalescent serum compared to the acute phase, and (iv) acute-phase serum positive for dengue virus-specific nonstructural glycoprotein-1 (NS1) antigen (Bio-Rad Laboratories, Marnes-la-Coquette, France) [22],[23]. Dengue diagnostic tests were performed by the Centers for Disease Control and Prevention in Taiwan. Dengue patients in this study were classified as dengue fever without warning signs, dengue fever with warning signs and severe dengue based on 2009 WHO case definitions [7]. Patients who did not meet the laboratory diagnosis criteria for influenza and dengue were classified as OFI in our analyses.

Leukopenia was defined as a peripheral white cell count <3.0 × 10^9^ cells/L (reference value, 3.0-10.5 × 10^9^ cells/L), mild thrombocytopenia as a peripheral platelet count of 149-100 × 10^9^ cells/L, and marked thrombocytopenia as a peripheral platelet count <100 × 10^9^ cells/L. Acute hepatitis referred to serum alanine aminotransferase (ALT) levels greater than 1,000 U/L (reference value, <40 U/L).

### Statistical analysis

Patients were categorized as having (i) influenza, (ii) dengue, or (iii) OFI. To distinguish influenza from dengue or OFI in a dengue and non-dengue endemic settings, univariate analyses were performed to compare clinical and laboratory characteristics between (i) influenza and dengue, and (ii) influenza and OFI. Categorical variables were compared using Chi-square or Fisher exact tests, and continuous variables were compared using Student's *t* or Mann-Whitney U tests. A 2-tailed P <0.05 was considered statistically significant. Significant variables in the univariate analyses were entered into a multivariate logistic regression model to determine independent predictor(s) of (i) influenza versus dengue, and (ii) influenza versus OFI. The sensitivity and specificity of each significant variable was assessed. Classification and regression tree analysis was performed [24]. The variables found to be significant in multivariate logistic regression with sensitivity greater than 80% were used to establish diagnostic decision trees to distinguish between patients with influenza and those with dengue [24]. Diagnosis was achieved by stepwise binary splitting, where one variable was entered at each node and, depending on the answer, a branch of the tree containing another variable was followed. Splitting stopped at nodes with a minimum classification of dengue, maximum classification of influenza, or a small number of patients in the node. For each terminal node, patients were classified as having low or high probability for influenza infection. To investigate the impact of age on the clinical presentation of influenza, dengue, and OFI, stratified analyses were performed for pediatric (≤18 years) and adult (>18 years) patients to examine the differences in clinical and laboratory characteristics between (i) influenza and dengue, and (ii) influenza and OFI.

## Results

### Demographic and clinical features of the patients

Of 849 patients, 526 (315 men and 211 women; mean age, 13.6 ± 13.5 years) were diagnosed with influenza, 148 (83 men and 65 women; mean age, 45.1 ± 4.5 years) with dengue, and 175 (106 men and 69 women; mean age, 15.3 ± 15.9 years) with OFI. None of the patients had concurrent influenza and dengue infections. Demographic, clinical, and laboratory information are summarized in Tables 1 and 2.

**Table 1:** **Demographics**, **clinical features**, **and outcomes of patients with influenza**, **dengue**, **and other febrile illnesses**

Variable	Influenza (n = 526)	Dengue (n = 148)	Other febrile illnesses (n = 175)	P ^a^	P ^b^
Mean age (± SD), years	13.6 ± 13.5	45.1 ± 4.5	15.3 ± 15.9	<0.001	0.941
Age group (n, [%])					
≤ 18 years	445 (84.6)	24 (16.2)	130 (74.3)	-	-
>18 years	81 (15.4)	124 (83.8)	45 (25.7)	-	-
Male gender (n, [%])	315 (59.9)	83 (56.1)	106 (60.6)	0.406	0.873
Underlying condition^c^ (n, [%])					
Bronchial asthma	14 (26.7)	0	5 (2.9)	-	-
Diabetes mellitus	10 (1.9)	17 (11.5)	2 (1.1)	-	-
Hypertension	15 (2.8)	30 (20.3)	2 (1.1)	-	-
Influenza A virus subtype (n, [%])					
Pandemic 2009 H1N1	447 (85)	-	-	-	-
Seasonal H3N2	79 (15)	-	-	-	-
Dengue disease severity (n, [%])					
Dengue without warning signs	-	64 (43.2)	-	-	-
Dengue with warning signs	-	77 (52)	-	-	-
Severe dengue	-	7 (4.7)	-	-	-
Dengue virus serotypes (n/N, [%])					
Serotype I	-	10/40 (25)	-	-	-
Serotype II	-	17/40 (42.5)	-	-	-
Serotype III	-	12/40 (30)	-	-	-
Serotype IV	-	1/40 (2.5)	-	-	-
Mean interval from onset of symptoms to emergency room presentation, days (± SD)	1.7 ± 1.2	3.7 ± 1.8	3.0 ± 2.1	-	-
Mean fever duration, days (± SD)	2.8 ± 1.6	4.0 ± 2.3	ND	<0.001	-
Use of oseltamivir >48 h (n/N, [%])	107/364 (29.4)	-	-	-	-
Fatalities (n, [%])	5 (0.9)	0	0	0.234	0.196

n/N = number of patients/total number of patients with data available; ND = no data.

^a^Influenza vs. dengue.

^b^Influenza vs. other febrile illnesses.

^c^An individual patient might have more than one underlying disease/condition.

**Table 2:** **Symptoms**/**signs and laboratory characteristics of patients with influenza**, **dengue**, **and other febrile illnesses**

Variable	Influenza (n = 526)	Dengue (n = 148)	Other febrile illnesses (n = 175)	P ^a^	P ^b^
Symptom/sign^c^ (n [%])					
Rhinorrhea	333 (63.3)	0	73 (41.7)	<0.001	<0.001
Cough	356 (67.6)	38 (25.7)	112 (64)	<0.001	0.396
Sore throat	207 (39.3)	24 (16.2)	43 (24.6)	<0.001	<0.001
Malaise	183 (34.8)	95 (64.2)	21 (12)	<0.001	<0.001
Headache	149 (28.3)	62 (41.9)	33 (18.9)	0.001	0.015
Vomiting/nausea	116 (22.1)	43 (29.1)	37 (21.1)	0.076	0.801
Diarrhea	57 (10.8)	15 (10.1)	26 (14.9)	0.807	0.154
Abdominal pain	40 (7.6)	31 (20.9)	18 (10.3)	<0.001	0.265
Joint pain	26 (4.9)	74 (50)	10 (5.7)	<0.001	0.539
Orbital pain	0	17 (11.5)	2 (1.1)	<0.001	0.062
Rashes	5 (0.9)	88 (59.5)	0	<0.001	0.196
Dyspnea	41 (7.8)	3 (2)	1 (0.6)	0.008	<0.001
Drowsiness	13 (2.5)	0	0	0.083	0.036
Seizures	8 (1.5)	0	0	0.211	0.211
Laboratory characteristics					
Leukopenia (WBC <3.0 × 10^9^ cells/L) (n/N [%])	9/283 (3.2)	82/146 (56)	4/152 (2.6)	<0.001	0.749
Platelet count 149-100 × 10^9^ cells/L (n/N [%])	38/238 (15.9)	27/146 (18.5)	5/133 (3.7)	0.522	0.003
Platelet count <100 × 10^9^ cells/L (n/N [%])	7/238 (2.9)	109/146 (74.6)	3/133 (2.3)	<0.001	0.696

WBC = white blood cell count; n/N = number of patients/total number of patients with data available.

^a^Influenza vs. dengue.

^b^Influenza vs. other febrile illnesses.

^c^An individual patient might have more than one symptom/sign.

Of 526 (445 [84.6%] patients aged ≤18 years) influenza infections, 447 (85%) were 2009 pandemic H1N1 and 79 (15%) were seasonal H3N2. The mean interval between onset of illness and the patient's arrival at the ER was 1.7 ± 1.2 days, and the mean fever duration was 2.8 ± 1.6 days. Besides fever, the 3 most common symptoms were cough (67.6%), rhinorrhea (63.3%), and sore throat (39.3%). Mild thrombocytopenia was detected in 38 (15.9%) of the 238 patients with an available peripheral platelet count. Of 364 patients receiving oseltamivir, 107 (29.4%) started therapy >48 h after illness onset. The 3 most common influenza-associated complications were pneumonia (5.1%), meningoencephalitis (1.1%), and acute respiratory distress syndrome (ARDS) (0.9%) (Table 3). The mortality rate was 0.9%, comprised of 5 patients (2 pandemic 2009 H1N1 and 3 seasonal H3N2; 4 women and 1 man, median age of 24 years [range 2-72 years]). All fatal cases received oseltamivir therapy >48 h after illness onset. Pneumonia was detected in all 5 fatal cases, ARDS and meningoencephalitis each in 3 (60% each), intracranial hemorrhage in 2 (40%), and myocarditis and gastrointestinal bleeding each in 1 (20% each).

**Table 3:** **Complications of influenza and dengue infections**

Complication ^a^	Influenza (n = 526)	Dengue (n = 148)	P
Pneumonia	27^b^ (5.1)	1 (0.6)	0.016
ARDS	5^c^ (0.9)	0	0.234
Meningoencephalitis	6^d^ (1.1)	0	0.192
Intracranial bleeding	2^d^ (0.4)	0	0.452
Gastrointestinal bleeding	2^d^ (0.4)	14 (9.5)	<0.001
Myocarditis	1^d^ (0.2)	0	0.596
Acute hepatitis (ALT >1,000 U/L)	2^d^ (0.4)	0	0.452

Data are number (%) of patients. ARDS = acute respiratory distress syndrome; ALT = alanine aminotransferase.

^a^An individual patient might have more than one complication.

^b^Of 27 influenza patients with pneumonia, 15 cases were 2009 pandemic H1N1 and 12 cases were H3N2.

^c^Of 5 influenza patients with ARDS, 4 cases were 2009 pandemic H1N1 and 1 case was H3N2.

^d^Patient(s) with 2009 pandemic H1N1 infection.

Among 148 patients diagnosed with dengue infection (24 [16.2%] patients aged ≤18 years), 64 (43.2%) were dengue fever without warning signs, 77 (52%) were dengue fever with warning signs, and 7 (4.7%) were severe dengue, based on 2009 WHO case definitions [7]. An individual patient might have received more than one dengue diagnostic test. Of laboratory-positive dengue cases, 40 were confirmed by RT-PCR, 71 by IgM of acute phase serum, 47 by fourfold increase in IgG titer in paired acute and convalescent serum, and 78 by detection of NS1 antigen. Of 71 patients positive for IgM antibody, a fourfold rise in IgG titer in paired serum was found in 31 patients, detection of NS1 antigen in 29, positive RT-PCR and NS1 antigen in 6, fourfold increase in IgG titer in paired serum and positive NS1 antigen in 3, and fourfold rise in IgG titer in paired serum and positive RT-PCR in 2. The mean time lapse from onset of symptoms to ER presentation was 3.7 ± 1.8 days, and the mean fever duration was 4.0 ± 2.3 days. The 3 most common symptoms other than fever in dengue patients were malaise (64.2%), rashes (59.5%), and joint pain (50%). Marked thrombocytopenia and leukopenia were found in 109 (74.6%) and 82 (56%) patients, respectively, of 146 patients with complete blood counts. Forty patients had available RT-PCR data; of these, dengue virus serotype II was detected in 17 (42.5%) patients, serotype III in 12 (30%), serotype I in 10 (25%), and serotype IV in 1 (2.5%). Of 148 dengue patients, 14 (9.5%) experienced gastrointestinal bleeding (Table 3). All dengue patients recovered.

Table 4 describes the diagnosis of the 175 patients with OFI (130 [74.3%] patients aged ≤18 years). The 3 leading etiologies of OFI were acute pharyngitis (38.8%), acute bronchitis (25.7%), and bronchopneumonia (16%). The mean interval from onset of illness to ER presentation was 3.0 ± 2.1 days. All patients with OFI recovered.

**Table 4:** **Etiologies of 175 patients with other febrile illnesses**

Variable	
Acute pharyngitis	68 (38.8)
Acute bronchitis	45 (25.7)
Bronchopneumonia	28 (16)
Acute tonsillitis	16 (9.1)
Gastroenteritis	11 (6.3)
Sinusitis	2 (1.1)
Kawasaki	1 (0.5)
Unknown	4 (2.3)

Data are number (%) of patients.

### Clinical and laboratory characteristics distinguishing influenza from dengue

Comparisons between patients with influenza (n = 526) and dengue (n =148) are shown in Tables 1, 2, 3, 5, and 6. Patients with influenza were significantly younger, had shorter fever duration, and presented at the ER earlier than patients with dengue (Table 1). Rhinorrhea, cough, sore throat, and dyspnea were reported significantly more frequently in patients with influenza than those with dengue (Table 2). Significantly lower frequencies of malaise, headache, abdominal pain, joint pain, orbital pain, and rashes were noted in patients with influenza than in those with dengue (Table 2). Dengue patients had significantly higher incidences of leukopenia, marked thrombocytopenia, and gastrointestinal bleeding in addition to lower incidence of pneumonia (Tables 2 and 3). Multivariate analysis disclosed absence of rashes (odds ratio [OR], 131.336), no leukopenia (OR, 24.978), and no marked thrombocytopenia (OR, 105.973) as predictive factors that distinguished influenza from dengue (Table 6). The sensitivities of absence of rashes, without leukopenia, and no marked thrombocytopenia were 98.3%, 96.9%, and 97.9%, while the specificities were 60%, 55.9%, and 74.5%, respectively.

**Table 5:** **Age**-**specific symptoms**/**signs and laboratory features of patients with influenza**, **dengue**, **and other febrile illnesses**

Variable	Age ≤18 years		Age >18 years	
	Inf vs. Den (Inf, n = 445; Den, n = 24)	Inf vs. OFI (Inf, n = 445; OFI, n = 130)	Inf vs. Den (Inf, n = 81; Den, n = 124)	Inf vs. OFI (Inf, n = 81; OFI, n = 45)
Symptom/sign^a^ (n [%])				
Rhinorrhea	297 (66.7) vs. 0***	297 (66.7) vs. 65 (50)**	36 (44.4) vs. 0***	36 (44.4) vs. 8 (17.8)**
Cough	311 (69.9) vs. 3 (12.5)***	311 (69.9) vs. 94 (72.3)	45 (55.6) vs. 35 (28.2)***	45 (55.6) vs. 18 (40)*
Sore throat	163 (36.6) vs. 4 (16.7)	163 (36.6) vs. 27 (20.8)**	44 (54.3) vs. 20 (16.1)***	44 (54.3) vs. 16 (35.6)
Malaise	141 (31.6) vs. 14 (58.3)*	141 (31.6) vs. 8 (6.2)***	42 (51.9) vs. 81 (65.3)	42 (51.9) vs. 13 (28.9)*
Headache	125 (28.1) vs. 11 (45.8)	125 (28.1) vs. 21 (16.2)**	24 (29.6) vs. 51 (41.1)	24 (29.6) vs. 12 (26.7)
Vomiting/nausea	106 (23.8) vs. 8 (33.3)	106 (23.8) vs. 33 (25.4)	10 (12.3) vs. 35 (28.2)**	10 (12.3) vs. 4 (8.9)
Diarrhea	54 (12.1) vs. 3 (12.5)	54 (12.1) vs. 19 (14.6)	3 (3.7) vs. 12 (9.7)	3 (3.7) vs. 7 (15.6)*
Abdominal pain	34 (7.6) vs. 4 (16.7)	34 (7.6) vs. 14 (10.8)	6 (7.4) vs. 27 (21.8)**	6 (7.4) vs. 4 (8.9)
Joint pain	18 (4) vs. 6 (25)	18 (4) vs. 7 (5.4)	8 (9.9) vs. 68 (54.8)***	8 (9.9) vs. 3 (6.7)
Orbital pain	0 vs. 2 (8.3)**	0 (0) vs. 2 (1.5)	0 vs. 15 (12.1)**	0 vs. 0
Rashes	5 (1.1) vs. 11(45.8)***	5 (1.1) vs. 0	0 vs. 77 (62.1)***	0 vs. 0
Dyspnea	24 (5.6) vs. 1 (4.1)	24 (5.6) vs. 1 (0.8)*	17 (3.7) vs. 2 (1.6)***	17 (3.7) vs. 0**
Drowsiness	10 (2.2) vs. 0	10 (2.2) vs. 0	3 (3.7) vs. 0	3 (3.7) vs. 0
Seizures	6 (1.3) vs. 0	6 (1.3) vs. 0	2 (2.5) vs. 0	2 (2.5) vs. 0
Laboratory characteristics				
Leukopenia (WBC <3.0 × 10^9^ cells/L) (n/N [%])	6/231 (2.6) vs. 14/23 (60.8)***	6/231 (2.6) vs. 1/116 (0.8)	3/52 (5.8) vs. 68/123 (55.3)***	3/52 (5.8) vs. 3/36 (8.3)
Platelet count 149-100 × 10^9^ cells/L (n/N [%])	24/189 (12.6) vs. 6/23 (26)	24/189 (12.6) vs. 2/106 (1.9)**	14/49 (28.6) vs. 21/123 (17.1)	14/49 (28.6) vs. 3/27 (11.1)
Platelet count <100 × 10^9^ cells/L (n/N [%])	3/189 (1.6) vs. 12/23 (52.2)***	3/189 (1.6) vs. 2/106 (1.9)	4/49 (8.2) vs. 97/123 (78.9)***	4/49 (8.2) vs. 1/27 (3.7)

Inf = influenza; Den = dengue; OFI = other febrile illnesses; WBC = white blood count; n/N = number of patients/total number of patients with data available.

*P < 0.05; **P < 0.01; ***P < 0.001.

^a^An individual patient might have more than one symptom/sign.

**Table 6:** **Sensitivity**, **specificity**, **and multivariate logistic regression for prediction of influenza versus dengue and influenza versus other febrile illnesses**

	Influenza n/N (%)	Dengue n/N (%)	Other febrile illnesses n/N (%)	Adjusted odds ratio	95% CI	P	Sensitivity%	Specificity%
**Influenza vs. dengue**								
All ages								
No skin rashes	234/238 (98.3)	58/145 (40)	-	131.336	35.416-487.039	<0.001	98.3	60
No leukopenia^a^	225/232 (96.9)	64/145 (44.1)	-	24.978	7.010-89.006	<0.001	96.9	55.9
No marked thrombocytopenia^b^	233/238 (97.9)	37/145 (25.5)	-	105.973	31.687-354.419	<0.001	97.9	74.5
Age ≤18 years								
No skin rashes	185/189 (97.9)	12/23 (52.2)	-	326.393	30.860-3452.098	<0.001	97.9	52.2
No leukopenia^a^	185/189 (97.9)	9/23 (39.1)	-	122.116	10.888-1369.665	<0.001	97.9	60.9
No marked thrombocytopenia^b^	186/189 (98.4)	11/23 (47.8)	-	88.632	6.443-1219.174	0.001	98.4	52.2
Age >18 years								
No leukopenia^a^	46/49 (93.9)	55/122 (45.1)	-	13.99	3.628-53.946	<0.001	93.9	54.9
No marked thrombocytopenia^b^	45/49 (91.8)	26/122 (21.3)	-	34.096	10.622-109.445	<0.001	91.8	78.7
**Influenza vs. other febrile illnesses**								
All ages								
Rhinorrhea	152/238 (63.9)	-	57/137 (41.6)	3.350	1.997-5.618	<0.001	63.9	58.4
Malaise	105/238 (44.1)	-	16/137 (11.7)	6.050	3.207-11.414	<0.001	44.1	88.3
Sore throat	105/238 (44.1)	-	30/137 (21.9)	3.407	1.960-5.922	<0.001	44.1	78.1
Dyspnea	41/238 (17.3)	-	1/137 (0.7)	47.335	6.174-632.919	<0.001	17.3	99.3
Mild thrombocytopenia^c^	38/238 (15.9)	-	5/137 (3.6)	3.779	1.309-10.908	0.014	15.9	96.4
Age ≤18 years								
Rhinorrhea	131/189 (69.3)	-	53/106 (50)	2.765	1.531-4.997	0.001	69.3	50
Malaise	76/189 (40.2)	-	6/106 (5.7)	11.129	4.455-27.802	<0.001	40.2	94.3
Sore throat	83/189 (43.9)	-	21/106 (19.8)	3.575	1.892-6.753	<0.001	43.9	80.2
Dyspnea	24/189 (12.7)	-	1/106 (0.9)	20.867	2.641-164.872	0.004	12.7	99
Mild thrombocytopenia^c^	24/189 (12.7)	-	2/106 (1.9)	7.138	1.509-33.769	0.013	12.7	98.1
Age >18 years		-						
Rhinorrhea	36/81 (44.4)	-	8/45 (17.8)	4.726	1.654-13.5	0.004	44.4	82.2
Malaise	42/81 (51.9)	-	13/45 (28.9)	3.108	1.224-7.897	0.017	51.9	71.1

n/N = number of patients/total number of patients with data available; CI = confidence interval.

^a^Leukopenia is defined as white cell counts <3.0 × 10^9^ cells/L.

^b^Marked thrombocytopenia is defined as platelet counts <100 × 10^9^ cells/L.

^c^Mild thrombocytopenia is defined as platelet counts 149-100 × 10^9^ cells/L.

Symptoms and laboratory characteristics of influenza infection varied by patient age (Table 5). Pediatric influenza patients (≤18 years, n = 445) had significantly higher frequencies of rhinorrhea and cough; lower frequencies of malaise, orbital pain and rashes; and lower incidences of leukopenia and marked thrombocytopenia compared with dengue patients (n = 24) (Table 5). Multivariate analysis showed absence of rashes (OR, 326.393), no leukopenia (OR, 122.116) and no marked thrombocytopenia (OR, 88.632) to be independent predictors for distinguishing influenza from dengue in pediatric patients (Table 6). For individuals >18 years of age, patients with influenza (n = 81) had greater occurrence of rhinorrhea, cough, sore throat, and dyspnea; they also presented with lower frequencies of nausea and vomiting, abdominal pain, joint pain, orbital pain, and rashes as well as lower incidences of leukopenia and marked thrombocytopenia (Table 5) compared to patients with dengue (n = 124). Multivariate analysis indicated that no leukopenia (OR, 13.99) and no marked thrombocytopenia (OR, 34.096) distinguished influenza from dengue in adults (Table 6).

### Clinical and laboratory characteristics distinguishing influenza from OFI

Comparisons between patients with influenza (n = 526) and OFI (n = 175) are summarized in Tables 1, 2, 5, and 6. Significant differences in clinical and laboratory features between influenza and OFI included the presence of rhinorrhea, sore throat, malaise, headache, dyspnea, drowsiness, and mild thrombocytopenia (Table 2). Multivariate analysis revealed that rhinorrhea (OR, 3.350), malaise (OR, 6.050), sore throat (OR, 3.407), dyspnea (OR, 47.335), and mild thrombocytopenia (OR, 3.779) were independent predictive factors that distinguished influenza from OFI (Table 6). The sensitivities of rhinorrhea, malaise, sore throat, dyspnea, and mild thrombocytopenia were 63.9%, 44.1%, 44.1%, 17.3%, and 15.9%, respectively.

As shown in Tables 5 and 6, significantly higher proportions of pediatric patients with influenza (n = 445) experienced rhinorrhea, sore throat, malaise, headache, dyspnea, and mild thrombocytopenia compared with OFI (n = 130); multivariate analysis showed rhinorrhea (OR, 2.765), malaise (OR, 11.129), sore throat (OR, 3.575), dyspnea (OR, 20.867), and mild thrombocytopenia (OR, 7.138) to be independent predictive factors that differentiated influenza from OFI in pediatric patients. Compared to adults with OFI (n = 45), adult patients with influenza (n = 81) had significantly higher frequencies of rhinorrhea, cough, malaise, diarrhea, and dyspnea; multivariate analysis revealed rhinorrhea (OR, 4.726) and malaise (OR, 3.108) to be predictive factors that distinguished influenza from OFI in adults.

### Classification tree distinguishing influenza from dengue

**Figure 1:**
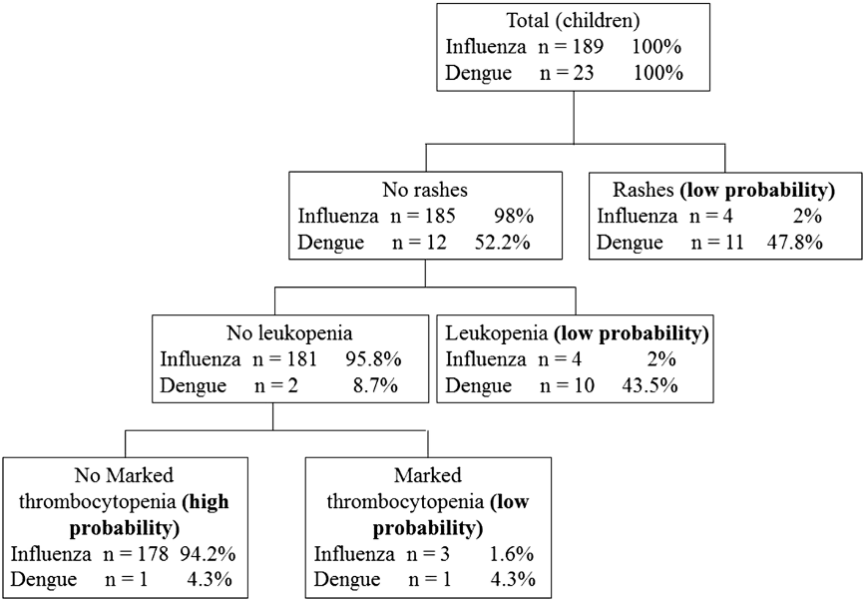
**Diagnostic algorithm to discriminate between influenza and dengue in pediatric patients.** Terminal nodes are marked as "low probability" or "high probability" for influenza infection.

**Figure 2:**
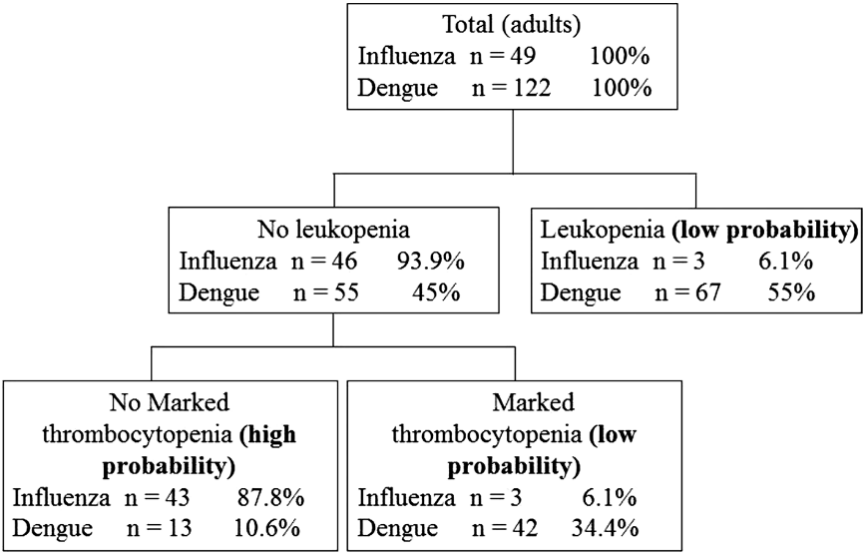
**Diagnostic algorithm to discriminate between influenza and dengue in adult patients.** Terminal nodes are marked as "low probability" or "high probability" for influenza infection.

Variables found to be significant in multivariate logistic regression were used to establish classification and regression trees. As shown in Figure 1, the initial splitting variable in the tree is a rash, followed by leukopenia and marked thrombocytopenia to distinguish influenza from dengue. The three nodes with low probability of influenza infection in pediatric patients were (i) presence of rashes, (ii) absence of rashes but with leukopenia and (iii) absence of rashes, without leukopenia, but with marked thrombocytopenia. There was a high probability of influenza infection among pediatric patients without rashes, no leukopenia and with no marked thrombocytopenia (94.2% of patients with influenza). Among adult patients, high probability of influenza was associated with no leukopenia and no marked thrombocytopenia (87.8% of patients with influenza) (Figure 2).

## Discussion

Our study investigated simple clinical and laboratory features to identify patients with influenza among children and adults with acute febrile illness in ER in dengue and non-dengue endemic areas. In our series, the mean time lapse between onset of illness and ER presentation was 1.7, 3.7, and 3 days for influenza, dengue, and OFI, respectively. This permits a detailed comparison of early clinical and laboratory characteristics between influenza and dengue or OFI.

The symptoms of influenza overlap substantially with dengue and OFI (Table 2). We found that symptoms resulting from respiratory tract infections such as cough, rhinorrhea, sore throat, and dyspnea were more prevalent in patients with influenza than with dengue. In contrast, non-respiratory tract symptoms including rashes, headache, abdominal pain, joint pain, and orbital pain were less common in the patients with influenza compared to patients with dengue. The influenza virus primarily infects epithelial cells of the respiratory tract and causes upper respiratory symptoms [25]. Damage to infected cells results in release of inflammatory mediators, leading to a systemic response (i.e., fever, headache, joint pain, malaise, and myalgia) with symptoms similar to other viral illnesses such as dengue [26],[27].

The overlapping geographic range of influenza and dengue as well as simultaneous dengue and influenza outbreaks [8]-[11], often in resource-limited countries, leads to diagnostic difficulties, as nonspecific symptoms are common to both infections. Early detection of influenza and dengue is especially important because influenza may be prevented through proper isolation and treated using antiviral agents [28],[29], whereas failure to make a timely dengue diagnosis with adequate fluid replacement can lead to severe dengue [30]. Our study demonstrated that the presence of leukopenia (white cell count <3.0 × 10^9^ cells/L) and marked thrombocytopenia (platelet count <100 × 10^9^ cells/L) are useful for differentiating dengue from influenza in both adults and pediatric patients. The absence of rash further discriminated influenza from dengue in pediatric patients. Rash associated with influenza is not a common manifestation, occurring in only 2-8% of patients, usually children [31],[32]. In contrast, the reported frequency of rash in dengue cases ranges from 46-68%, particularly in children less than 15 years who usually have a nonspecific febrile syndrome accompanied by rash [33],[34]. Mild leukopenia and relative lymphopenia are typical findings of influenza; thrombocytopenia may be present in complicated cases [4],[14],[25]. Notably, our study and others found leukopenia and thrombocytopenia to be common laboratory findings in dengue infection, with platelet counts below 20 × 10^9^ per liter often observed in severe dengue [7],[30]. A study examining predictors of diagnosis in 1,962 febrile travelers returning from the tropics found the main predictors of dengue infection compared with other fevers, excluding malaria, to include skin rash, thrombocytopenia, and leukopenia [35]. This finding was consistent with our study findings.

Our data suggest that skin rash, leukopenia (white cell count <3.0 × 10^9^ cells/L), and platelet counts <100 × 10^9^ cells/L were useful to predict negative influenza results during influenza and dengue epidemics. However, it is unlikely that any single indicator will be useful in clinical practice because these symptoms and laboratory findings can be present in both infections [11]. In our series, we developed two simple and practical diagnostic algorithms using clinical and laboratory indicators to distinguish influenza from dengue in adult and pediatric patients, respectively. We found that the diagnostic algorithm correctly classified 94.2% of pediatric patients with influenza in the "high probability" group with only one misclassified dengue patient (Figure 1). Analysis of our data after excluding pediatric patients showed similar results, except that skin rash that was no longer associated with influenza infection in adult patients; however, the diagnostic tree (Figure 2) still correctly classified 87.8% of adult influenza cases as "high probability". These findings underscored that rash, leukopenia, and marked thrombocytopenia could help to establish a diagnostic algorithm to distinguish influenza from dengue patients during outbreaks of both diseases. Additional prospective studies are needed to validate this predictive model in other dengue-endemic regions and in populations with different ethnicities.

The wide range of influenza-associated symptoms often makes it difficult to distinguish from other febrile or respiratory illnesses [25]. A crowded ER can make it challenging for physicians to differentiate between influenza and OFI [36]. Early antiviral treatment (≤48 h) of influenza is especially important for patients with underlying risk factors to avoid otherwise preventable morbidity and mortality [37]. Our results demonstrate that rhinorrhea, malaise, sore throat, and dyspnea in addition to a slightly low platelet count (100-149 × 10^9^ cells/L) in children, as well as rhinorrhea and malaise in adults were valuable predictors during the evaluation of the likelihood of influenza versus OFI in a non-dengue endemic setting. However, apart from rhinorrhea, which lacked adequate sensitivity and specificity, we found that all other variables were specific (>70%) but not sensitive enough in distinguishing influenza from OFI. This is not surprising as more than 60% of OFI cases included in this series were individuals with pharyngitis or bronchitis (Table 4), another viral infection commonly encountered in the ER [38]. The information of this study are most helpful for clinicians to facilitate diagnosis of influenza during periods of high influenza activity in non-dengue endemic setting.

In the present study, patients with influenza had a shorter fever duration than the dengue cases (mean 2.8 ± 1.6 vs. 4.0 ± 2.3 days). This finding is similar to a previous study that found a fever duration of <4 days for patients with influenza [25], while the average length of fever in dengue patients was approximately 5 days [39], coinciding with the disappearance of viremia.

Although influenza primarily causes upper respiratory tract infections, pulmonary and extra-pulmonary complications have also been reported [2],[40],[41]. Previous studies from the United States and Australia of critically ill patients with 2009 pandemic H1N1 infections found ARDS complication in 35.8% and 48.8% of cases and a 45% and 14.3% hospital mortality, respectively [40],[41]. ARDS was noted in 5 influenza cases in our study (Table 3); 3 were fatal. Remarkably, delayed oseltamivir therapy was found in all fatal cases in our series. The importance of a timely anti-viral therapy for severe influenza should therefore be emphasized.

Earlier studies describe neurological complications of influenza including aseptic meningitis, encephalopathy/encephalitis, Guillain-Barré syndrome, and transverse myelitis [42]-[44]. Neurological complications have been reported for patients with 2009 pandemic H1N1 infections [43]. Of 447 patients with 2009 pandemic H1N1 infection in this study, meningoencephalitis was found in 6 (1.3%), with presenting symptoms of altered mental status and seizures (Table 3). Other influenza-associated extrapulmonary complications among our patients included myocarditis, rhabdomyolysis, gastrointestinal bleeding, and intracranial hemorrhage (Table 3). Physicians should be aware of influenza-associated extrapulmonary complications when caring for influenza patients and manage them accordingly.

Gastrointestinal bleeding was the most common complication among dengue patients in this study. Previous studies have reported gastrointestinal bleeding to be a warning sign of severe dengue; timely management and intensive monitoring of patients with gastrointestinal bleeding is therefore necessary [45].

This study has several limitations. First, because it was conducted in a single medical center, disease severity may have been biased by referral patterns. Second, as a retrospective study, missing laboratory data was inevitable. Third, the decision to perform diagnostic tests for influenza and dengue infections was based on individual physicians' experience; therefore, patients included in our series may have been biased by individual physicians' personal judgments. In addition, our results were based on data from early stages of illness; future studies are necessary to validate these findings in different course of illness for better generalization of their utility.

## Conclusions

This study demonstrates substantial overlap in clinical presentation between influenza and dengue as well as OFI. This study also identifies simple and useful clinical and laboratory data to enable identification and facilitate diagnosis of influenza in different clinical settings (dengue and non-dengue endemic areas). We provide two decision tree algorithms using simple clinical and laboratory data that can be easily implemented in resource-limited countries to differentiate patients with influenza from those with dengue. This information is especially important to clinicians in countries where medical resources are sparse and the burden of influenza and dengue is high.

## Authors' contributions

SYH and IKL made major contributions to sample collection and wrote the manuscript. IKL conceived and designed the study, analysis of the data and preparation of the manuscript. SYH, SCH, CCC, TYC, WCH, JWL and LW collected clinical data. All authors read, commented on, and approved the final manuscript.

## Authors’ original submitted files for images

Below are the links to the authors’ original submitted files for images. Authors’ original file for figure 1 Authors’ original file for figure 2
