# Supplementary figures and images for: Use of simple clinical and laboratory predictors to differentiate influenza from dengue and other febrile illnesses in the emergency room
Source: BMC Infect Dis. 2014 Nov 25;14:623. doi: 10.1186/s12879-014-0623-z (PMC4245735; doi:10.1186/s12879-014-0623-z)

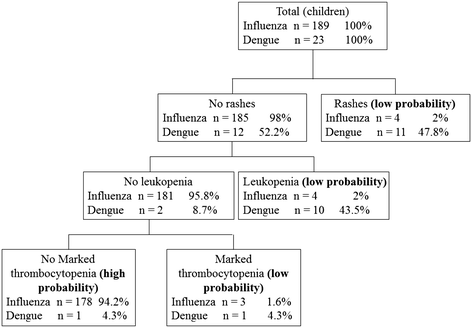

Supplement: Supplementary file 1 — Authors’ original file for figure 1 [file 12879_2014_623_MOESM1_ESM.gif]

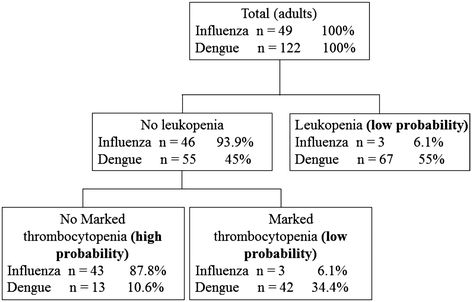

Supplement: Supplementary file 2 — Authors’ original file for figure 2 [file 12879_2014_623_MOESM2_ESM.gif]
